# Context-induced relapse after extinction versus punishment: similarities and differences

**DOI:** 10.1007/s00213-018-4929-1

**Published:** 2018-05-24

**Authors:** Nathan J. Marchant, Erin J. Campbell, Yann Pelloux, Jennifer M. Bossert, Yavin Shaham

**Affiliations:** 1 Department of Anatomy & Neurosciences, VU University Medical Center, Amsterdam, The Netherlands; 2 The Florey Institute of Neuroscience and Mental Health, Parkville, Victoria 3052 Australia; 3 Florey Department of Neuroscience and Mental Health, The University of Melbourne, Melbourne, Victoria 3010 Australia; 4 Behavioral Neuroscience Branch, Intramural Research Program, National Institute on Drug Abuse, Baltimore, MD USA

**Keywords:** Context, Craving, Punishment, Extinction, Drug self-administration, Reinstatement, Relapse, Review

## Abstract

Results from clinical studies suggest that drug relapse and craving are often provoked by exposure to drug-associated contexts. Since 2002, this phenomenon has been modeled in laboratory animals using the ABA renewal model. In the classical version of this model, rats with a history of drug self-administration in one context (A) undergo extinction in a different context (B) and reinstate (or relapse to) drug seeking after exposure to the original drug-associated context (A). In a more recent version of the model introduced in 2013, the experimental conditions in context A are identical to those used in the classical model, but drug-reinforced responding in context B is suppressed by probabilistic punishment. The punishment-based ABA renewal model is proposed to resemble abstinence in humans, which is often initiated by the desire to avoid the negative consequences of drug use. The goal of our review is to discuss similarities and differences in mechanisms that play a role in suppression of drug seeking in context B and context-induced relapse to drug seeking in context A in the two models. We first describe psychological mechanisms that mediate extinction and punishment of drug-reinforced responding in context B. We then summarize recent findings on brain mechanisms of context-induced relapse of drug seeking after extinction, or punishment-imposed abstinence. These findings demonstrate both similarities and differences in brain mechanisms underlying relapse in the two variations of the ABA renewal model. We conclude by briefly discussing clinical implications of the preclinical studies.

## Introduction

A major obstacle in the treatment of drug addiction is relapse to drug use after periods of abstinence (Hunt et al. 1971; Sinha 2011). In former drug users, drug craving and relapse during abstinence are often triggered by environments or contexts that were previously associated with drug use (O’Brien et al. 1992; Wikler 1973). This clinical scenario has been modeled in laboratory rats by using a variation of the extinction-reinstatement model (Bossert et al. 2013; de Wit and Stewart 1981; Shaham et al. 2003) that is based on the ABA renewal model (Bouton and Bolles 1979). In the classical ABA renewal model (also termed context-induced reinstatement), rats with a history of drug self-administration in one context (A) undergo operant extinction in a different context (B) and reinstate (or relapse to) drug seeking in context A (Crombag et al. 2008). The operational definition of reinstatement or relapse in the model is significantly higher non-reinforced operant responding in the original drug self-administration training context A than in the extinction context B (Crombag et al. 2008). Since the initial demonstration with speedball (a heroin-cocaine combination) (Crombag and Shaham 2002), context-induced reinstatement (or relapse) of drug seeking after extinction-induced abstinence has been observed with heroin (Bossert et al. 2004), cocaine (Crombag et al. 2002; Fuchs et al. 2005), alcohol (Chaudhri et al. 2008; Hamlin et al. 2007), nicotine (Diergaarde et al. 2008), and methamphetamine (Rubio et al. 2015; Widholm et al. 2011).

From a translational perspective, however, one important aspect of drug addiction that extinction does not model is the negative consequences of drug use. Specifically, human abstinence rarely involves lack of drug availability or overt extinction of drug-seeking responses (Epstein et al. 2006; Katz and Higgins 2003; Marlatt 2002). Instead, abstinence is typically initiated while the drug is available because of the desire to avoid the negative consequences associated with excessive drug use (Epstein and Preston 2003). Based on these considerations, we recently developed a context-induced relapse model that incorporates the negative consequences of drug use, in which alcohol self-administration is suppressed by adverse consequences (probabilistic operant punishment) (Marchant et al. 2013a). This model is a modified ABA renewal procedure in which abstinence is achieved in context B, despite alcohol availability, by punishment with response-contingent electric footshock. Using this model, we have demonstrated context-induced relapse to both alcohol and cocaine seeking when rats were tested in context A after punishment-imposed abstinence in context B (Marchant et al. 2014, 2016; Marchant and Kaganovsky 2015; Pelloux et al. 2018a, b). Our model and findings extend previous research in the addiction field on the use of punishment procedures to model the negative consequences of drug seeking and drug use (Deroche-Gamonet et al. 2004; Marchant et al. 2013b; Panlilio et al. 2003; Pelloux et al. 2007; Vanderschuren et al. 2017; Vanderschuren and Everitt 2004; Wolffgramm and Heyne 1995).

In the extinction- and punishment-based ABA renewal models, the test conditions are identical. Drug seeking in both situations is induced by exposure to drug-associated contexts, and the tests occur under extinction conditions. However, the methods used to impose that abstinence are different. Extinction-imposed suppression of drug seeking occurs in the *absence* of the drug, while punishment-imposed suppression of drug seeking occurs in the *presence* of the drug. Thus, the psychological mechanisms that underlie these two processes are different, and because of this, context-induced relapse may also rely on different neuronal mechanisms.

In this review, we first discuss methodological and conceptual issues related to the study of extinction and punishment of operant responding. We then summarize recent findings on brain areas and circuits that play a role in context-induced relapse of drug seeking after extinction- and punishment-imposed abstinence. We conclude by discussing the clinical implications of the similarities and differences in mechanisms underlying relapse, as assessed in these two models.

## Extinction and punishment: methodological and conceptual considerations

In operant conditioning, extinction and punishment are examples of retroactive interference learning that decreases the behavioral expression of the original response-outcome (R-O) operant learning (Bouton 1993, 2000). One key feature of retroactive interference learning is that the expression of the learned behavior is context-dependent (Bouton 1993, 2002). Indeed, renewal of reward seeking is observed in the original training context (A) when extinction or punishment occurs in a different context (B) (Baker et al. 1991; Bouton and Schepers 2015; Marchant et al. 2013a; Nakajima et al. 2000). Thus, an important similarity between extinction and punishment is that they both involve context-dependent learning that modulates the expression of the original operant association.

An important difference between extinction and punishment is that during extinction, the subject must learn a new association in which the operant response now leads to no reward (no outcome). In contrast, during punishment, the subject learns about a new relationship between the original operant response and a second stimulus (footshock), which has opposite motivational valence to the original appetitive stimulus (i.e., food or drug). Therefore, the key differences between extinction and punishment are that punishment involves a continued presence of the appetitive stimulus (reward), and that there is an additional aversive stimulus in punishment (shock). This operational difference causes differences in the underlying psychological mechanisms that are responsible for controlling behavior.

In extinction, new learning occurs regarding the fact that the response no longer leads to the drug reward (the outcome), and drug seeking is reduced. Extensive research on Pavlovian conditioning has led to the proposal that the extinction context functions as an occasion setter (Holland 1992). In this configuration, the context gates the expression of different associations (i.e., either CS-US or CS-NoUS). Crombag et al. (2002) proposed that this mechanism may also apply to context-induced reinstatement of extinguished drug seeking. However, recently Todd, Bouton, and colleagues have demonstrated that in instrumental conditioning, the extinction context forms a direct inhibitory association with the operant response (Bouton and Todd 2014; Todd et al. 2014). To date, comparable studies have not been conducted in rats trained to perform drug-reinforced instrumental responses. However, it is possible that extinction of drug-reinforced responses is also mediated by a direct inhibitory association between the extinction context and the drug-reinforced response.

The learning that occurs during punishment can form both operant and Pavlovian associations (Jean-Richard-Dit-Bressel et al. 2018). Thus, despite the fact that shock delivery is contingent on the operant response (R-O association), the introduction of a new stimulus (footshock) can cause the formation of Pavlovian stimulus-outcome (S-O) associations (e.g., lever shock) (Jean-Richard-Dit-Bressel et al. 2018; Marchant et al. 2017). Because of this inherent confound, it is important to consider whether punishment-imposed abstinence is mediated by operant R-O punishment learning or whether Pavlovian S-O fear conditioning can account for the suppression of the operant response (Estes and Skinner 1941). In this regard, exposure to Pavlovian cues and contexts previously paired with shock can suppress ongoing operant responding (Bouton and Bolles 1979; Estes and Skinner 1941; Pickens et al. 2009). The distinction between R-O operant punishment and S-O fear conditioning is important because different neurobiological mechanisms mediate operant punishment and Pavlovian conditioned suppression (Jean-Richard-Dit-Bressel et al. 2018).

Evidence for operant R-O learning in punishment was demonstrated by Bolles et al. (1980). They showed that S-O (Pavlovian) fear learning occurs during the initial punishment session, while R-O (operant) learning emerges towards the end of the first session and is fully apparent during the next training session. Jean-Richard-Dit-Bressel and McNally (2015) confirmed this finding using a two-lever design with retractable levers. They found that punishment of the response on one lever causes moderate levels of freezing (an index of conditioned fear) to both levers during the initial punishment sessions. However, during subsequent sessions, freezing induced by the extension of the levers is reduced, responding on the punished lever is suppressed, and responding on the unpunished lever is increased. Additional evidence that R-O operant punishment mediates suppression of drug seeking in context B in the studies reviewed below is that non-contingent random shock exposure in context B, which is independent of lever pressing, has no effect on alcohol self-administration (Marchant et al. 2013a). Bouton and Schepers (2015) and Pelloux et al. (2018a) expanded on this finding using a yoked-shock design where the number and temporal distribution of shocks between the punished and yoked groups are identical. They found that yoked non-contingent shock exposure has no effect on food or cocaine self-administration in context B.

Together, the studies reviewed above indicate that Pavlovian S-O learning occurs during the initial operant punishment learning but its impact on operant responding is limited to early learning, and is likely to extinguish over time. The recent observations discussed above that contingent but not non-contingent shock selectively suppresses alcohol, food, and cocaine self-administration further indicate that punishment-imposed abstinence is primarily mediated through operant R-O associations in studies using the punishment-based ABA renewal model (Bouton and Schepers 2015; Marchant et al. 2013a; Pelloux et al. 2018a).

In conclusion, while both extinction and punishment lead to context-dependent suppression of drug seeking in context B and renewal of drug seeking in context A, there are important differences in the learning and psychological mechanisms that contribute to the suppression of the operant response in context B. A question for future research is whether there are also differences in the psychological mechanisms of renewal in context A after extinction versus punishment.

## Brain mechanisms of context-induced relapse after extinction and punishment

In this section, we review results on the similarities and differences between context-induced relapse after extinction- or punishment-imposed suppression of drug seeking. We focus on studies using rats trained to self-administer alcohol or cocaine, because published studies on mechanisms of context-induced relapse after punishment with other drugs of abuse do not exist. We do not discuss circuit-related results from other studies using the classical extinction-based ABA renewal model, which we have recently reviewed (Marchant et al. 2015).

**Fig. 1:**
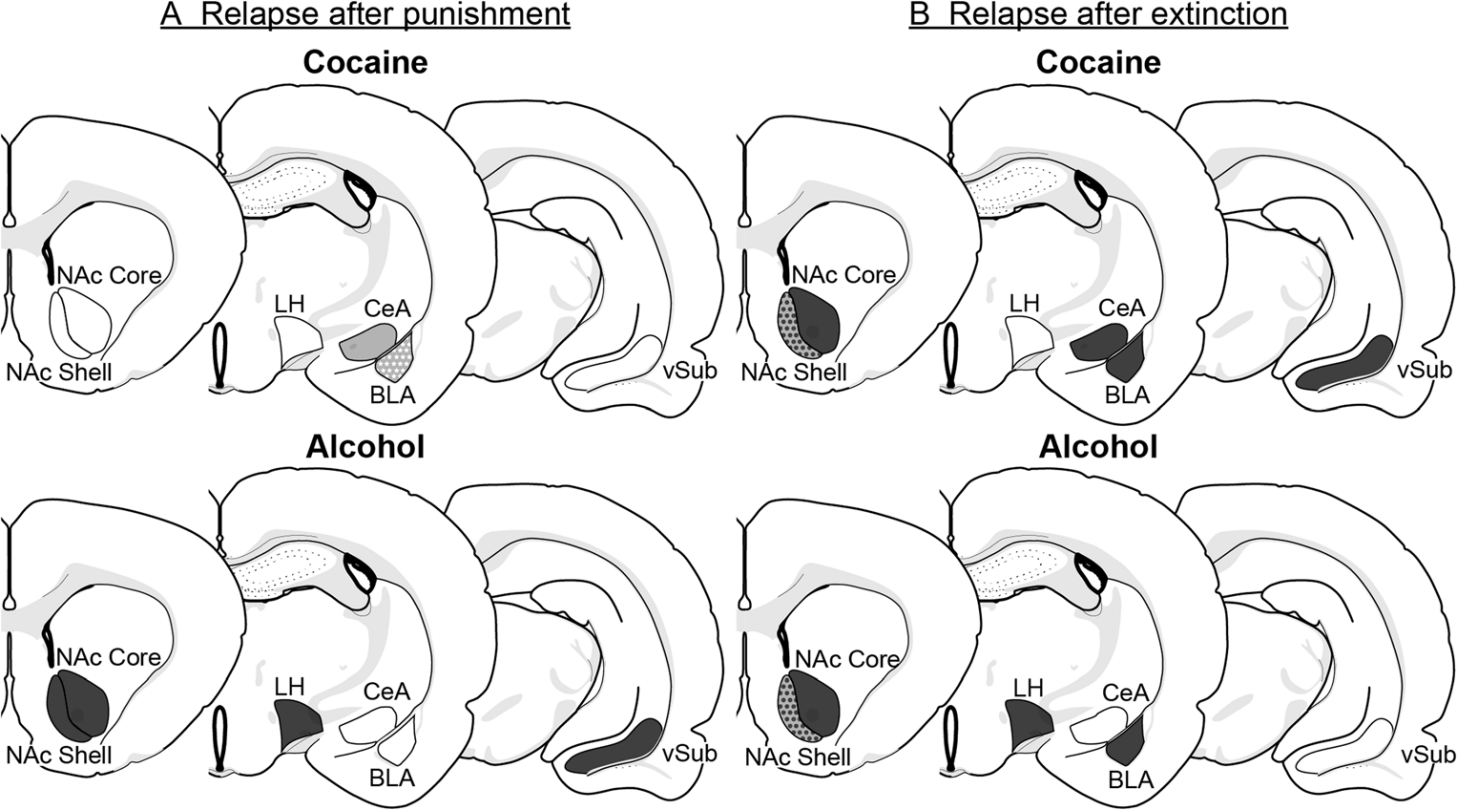
Effect of site-specific neuropharmacological manipulations on relapse to cocaine or alcohol seeking in context A or B after punishment- or extinction-imposed abstinence. **a** Manipulations in rats tested for context-induced relapse after punishment. **b** Manipulations in rats tested for context-induced relapse after extinction. Code: dark gray, decreased drug-seeking in context A; light gray, increased drug seeking in context B; white stars, increased drug-seeking in context A; white, either no effect or not tested. Abbreviations: BLA, basolateral amygdala; CeA, central amygdala; NAc, nucleus accumbens; LH, lateral hypothalamus; vSub, ventral subiculum

In Table 1, we provide correlational data from several studies in which we and other investigators have used the neuronal activity marker Fos (Cruz et al. 2013; Morgan and Curran 1991) to identify brain regions selectively activated during tests for context-induced relapse after extinction- or punishment-imposed abstinence. This table shows both similarities and differences in the regions activated during the relapse tests in the two models. We do not discuss the data described in Table 1 within the context of brain areas and circuits that play a role in context-induced relapse after extinction or punishment for two reasons. First, it cannot be ruled out that procedural differences related to the training, extinction, relapse test, and Fos assay conditions across the different studies can account for the observed differences in Fos expression (see Pelloux et al. (2018a) for a discussion of this issue). The second reason is that in the absence of follow-up functional causal role manipulations, correlational Fos data should be interpreted with caution. This is because Fos induction in different brain areas can reflect either the cause or the consequence of relapse to drug seeking and does not necessarily imply that a given brain area plays a causal role in relapse (Bossert et al. 2011; Cruz et al. 2013). In Table 2 and Fig. 1, we summarize the results on the effect of causal role neuropharmacological manipulations on context-induced relapse to drug seeking after extinction versus punishment, and discuss these data for each brain area.

**Table 1:** Comparison of Fos induction in different brain areas during the relapse tests in contexts A and B after either punishment- or extinction-imposed abstinence. Italics indicate a common effect in the two ABA renewal models

Brain region	Cocaine		Alcohol		References
	Punishment	Extinction	Punishment	Extinction	
dmPFC	A > B	A = B	A = B	A = B or A > B	Hamlin et al. (2007, 2008, 2009), Marchant et al. (2014), Palombo et al. (2017), Pelloux et al. (2018a), Perry and McNally (2013)
vmPFC	*A > B*	*A > B*	A = B	A = B or A < B	Hamlin et al. (2007, 2008, 2009), Marchant et al. (2010, 2014, 2016), Palombo et al. (2017), Pelloux et al. (2018a), Perry and McNally (2013)
DMS	A > B	A = B	A = B		Hamlin et al. (2008), Marchant et al. (2014), Pelloux et al. (2018a)
DLS	A > B	A = B	A > B		Hamlin et al. (2008), Marchant et al. (2014), Pelloux et al. (2018a)
NAc core	A = B	A = B	*A > B*	*A > B*	Cruz et al. (2014), Hamlin et al. (2008), Marchant et al. (2014), Pelloux et al. (2018a), Perry and McNally (2013)
NAc shell	A = B	A = B or A > B	A = B	A = B or A > B	Cruz et al. (2014), Hamlin et al. (2008), Marchant et al. (2009, 2014), Pelloux et al. (2018a), Perry and McNally (2013)
vBNST	A = B	A = B	A = B		Hamlin et al. (2008), Marchant et al. (2014), Pelloux et al. (2018a)
dBNST	A = B	A = B	A = B		Hamlin et al. (2008), Marchant et al. (2014), Pelloux et al. (2018a)
LS	A = B	A = B	A = B		Marchant et al. (2014), McGlinchey and Aston-Jones (2018), Pelloux et al. (2018a)
BLA	*A > B*	*A > B*	*A > B*	*A > B*	Hamlin et al. (2007, 2008, 2009), Marchant et al. (2016), Pelloux et al. (2018a), Perry and McNally (2013)
CeA	A = B	A = B			Hamlin et al. (2008), Pelloux et al. (2018a)
LHb	A > B		A < B		Marchant et al. (2014), Pelloux et al. (2018a)
MHb	A = B		A = B		Marchant et al. (2014), Pelloux et al. (2018a)
PVT	A > B	A = B	A = B or A > B	A = B	Hamlin et al. (2009), Marchant et al. (2014, 2016), Pelloux et al. (2018a), Perry and McNally (2013)
LH	A = B	A > B	*A > B*	*A > B*	Hamlin et al. (2007, 2008), Marchant et al. (2014), Pelloux et al. (2018a))
VTA	A = B	A = B		A = B	Hamlin et al. (2007, 2008), Pelloux et al. (2018a)
SN	A > B	A = B		A = B	Hamlin et al. (2007, 2008), Pelloux et al. (2018a)
vSub	A > B		A > B		Marchant et al. (2016), Pelloux et al. (2018a)
OFC	A = B			A > B	Pelloux et al. (2018a), Perry and McNally (2013)
AI	A > B			A = B	Hamlin et al. (2007), Pelloux et al. (2018a), Perry and McNally (2013)
VP	A = B			A > B	Pelloux et al. (2018a), Prasad and McNally (2016)

*AI*, anterior insular cortex; *BLA*, basolateral amygdala; *BNST*, bed nucleus of the stria terminalis; *CeA*, central amygdala; *dmPFC*, dorsomedial prefrontal cortex; *DMS*, dorsomedial striatum; *DH*, dorsal hippocampus; *DLS*, dorsolateral striatum; *LH*, lateral hypothalamus; *LHb*, lateral habenula; *LS*, lateral septum; *MHb*, medial habenula; *NAc*, nucleus accumbens; *OFC*, orbitofrontal cortex; *PVT*, paraventricular thalamus; *SN*, substantia nigra; *VH*, ventral hippocampus; *vmPFC*, ventromedial prefrontal cortex; *VP*, ventral pallidum; *VTA*, ventral tegmental area; *vSub*, ventral subiculum

**Table 2:** Effect of site-specific neuropharmacological manipulations on either cocaine or alcohol seeking during the relapse tests in either context A or B after either punishment- or extinction-imposed abstinence

Brain region	Pharmacological manipulation	Cocaine		Alcohol		References
		Punishment	Extinction	Punishment	Extinction	
LH	M+B			A↓, B–	A↓, B–	Marchant et al. (2009, 2014)
NAc core	M+B		A↓, B–		A↓, B–	Chaudhri et al. (2008, 2010), Fuchs et al. (2008)
NAc core	D1R			A↓, B–	A↓, B–	Chaudhri et al. (2009), Marchant and Kaganovsky (2015)
NAc shell	M+B		A↓, B↑		A↓ or A↕, B↑	Chaudhri et al. (2008, 2010), Fuchs et al. (2008), Millan et al. (2010)
NAc shell	D1R			A↓, B–	A↓, B–	Chaudhri et al. (2009), Marchant and Kaganovsky (2015)
VH/vSub	M+B		A↓, B–	A↓, B–		Lasseter et al. (2010), Marchant et al. (2016)
BLA	M+B	A↑, B↑	A↓, B–		A↓, B–	Chaudhri et al. (2013), Pelloux et al. (2018b)
CeA	M+B	A–, B↑	A↓, B–			Pelloux et al. (2018b)

–, no effect; ↓, decrease; ↑, increase; ↕, inconsistent results; *D1R*, D1-like receptor antagonist; *M+B*, muscimol+baclofen; *BLA*, basolateral amygdala; *CeA*, central amygdala; *NAc*, nucleus accumbens; *LH*, lateral hypothalamus; *VH*, ventral hippocampus; *vSub*, ventral subiculum

### Lateral hypothalamus

Results from two studies indicate that lateral hypothalamus (LH) activity is critical for context-induced relapse to alcohol seeking, independent of the method used to achieve abstinence. Reversible inactivation of LH with muscimol+baclofen (GABAa and GABAb agonists) decreases context-induced relapse to alcohol seeking after either extinction or punishment (Marchant et al. 2009, 2014). A question for future research is whether this putative general role of LH in context-induced relapse generalizes to other addictive drugs.

### Nucleus accumbens core

Results from several studies indicate a critical role of nucleus accumbens (NAc) core in context-induced relapse to alcohol seeking after either extinction or punishment. NAc core injections of muscimol+baclofen or the dopamine D1-family receptor antagonist SCH23390 decrease context-induced relapse to alcohol seeking after extinction, and NAc core SCH23390 injections decrease context-induced relapse after punishment (Chaudhri et al. 2008, 2010; Marchant and Kaganovsky 2015). It is currently unknown whether NAc core plays a similar role in context-induced relapse to cocaine seeking after punishment or extinction. Injections of muscimol+baclofen or glutamate receptors antagonists into NAc core decrease context-induced relapse to cocaine seeking after extinction (Fuchs et al. 2008; Xie et al. 2012), but see Cruz et al. (2014) for negative results using the Daun02 inactivation procedure in Fos-LacZ transgenic rats in which Daun02 injections into discrete brain areas selectively inactivate Fos-expressing neurons activated by exposure to drug-associated cues and contexts (Koya et al. 2009). The functional role of NAc core in context-induced relapse to cocaine seeking after punishment-imposed abstinence has not been investigated.

### Nucleus accumbens shell

NAc shell also appears to play a critical role in context-induced relapse of alcohol seeking after extinction or punishment. Injections of muscimol+baclofen or SCH23390 into NAc shell decrease context-induced relapse of alcohol seeking after extinction or punishment (Chaudhri et al. 2009; Marchant and Kaganovsky 2015). Context-induced relapse to alcohol seeking after extinction is also decreased by NAc shell injections of a mu-opioid receptor antagonist or the peptide cocaine-amphetamine-regulated transcript (CART) (Millan and McNally 2012; Perry and McNally 2013). Additionally, inhibition of the glutamatergic projection from ventral subiculum (vSub) to NAc shell decreases both context-induced relapse to alcohol seeking after punishment (Marchant et al. 2016) and context-induced relapse to heroin seeking after extinction (Bossert et al. 2016) using either a dual-virus approach to restrict expression of the inhibitory kappa opioid-receptor-based DREADD in vSub → NAc shell projection neurons or a pharmacological asymmetric disconnection procedure; in this procedure, neuronal activity of a given brain projection is inhibited by either injecting a drug that inhibits neuronal activity or by lesioning the cell body region in one hemisphere and the projection target in the other hemisphere (Gold 1966). Together, these results suggest that NAc shell activity is critical to context-induced relapse after either extinction or punishment across different drug classes.

However, NAc shell has also been implicated in the inhibition of extinguished alcohol and cocaine seeking (Millan et al. 2011; Peters et al. 2008). Specifically, while muscimol-baclofen inactivation of NAc shell or local injections of a glutamate AMPA receptor antagonist (NBQX) have no effect on context-induced relapse to alcohol seeking after extinction, these manipulations increase extinguished alcohol seeking in context B (Chaudhri et al. 2008; Millan and McNally 2011). Additionally, muscimol-baclofen inactivation of NAc shell induces reinstatement of cocaine or alcohol seeking after extinction in the drug self-administration context (Millan et al. 2010; Peters et al. 2008).

Taken together, the studies reviewed suggest a complicated role of NAc shell in regulating context-induced relapse to cocaine and alcohol seeking after extinction and an unexpected dissociation between dopamine- and glutamate-mediated neurotransmission in this form of relapse. This dissociation, however, does not generalize to heroin where inhibition of dopamine and glutamate transmission in NAc shell decreases context-induced relapse after extinction but has no effect on extinction responding in context B (Bossert et al. 2006, 2007). A recent study by Piantadosi et al. (2017) used food-trained rats to examine the role of NAc shell in mediating suppression of food seeking by punishment. They used a conflict design, where food reinforcement was first unpunished, and then punished, followed by another unpunished period. They found that NAc shell inactivation decreased food seeking during the unpunished periods, but increased food seeking during punishment. Given these findings, and in addition to the reinstatement-related findings reviewed above, we suspect that NAc shell activity will also play a complicated role in context-induced relapse after punishment that will depend on the neurotransmitter system and the neuropharmacological manipulation.

### Hippocampus

Given the role of hippocampus in mediating context-dependent functions, it is perhaps unsurprising that there have been several demonstrations that it is critical for context-induced relapse. Muscimol+baclofen inactivation of the dorsal hippocampus (DH) has been shown to decrease context-induced reinstatement of extinguished cocaine seeking (Fuchs et al. 2005). Furthermore, using the pharmacological disconnection procedure, via asymmetrical, unilateral inactivation of DH and BLA, Fuchs et al. (2007) showed that context-induced relapse of extinguished cocaine seeking is dependent on interaction between DH and BLA. In the ventral hippocampus (VH), muscimol+baclofen inactivation also decreases context-induced relapse of extinguished cocaine seeking (Lasseter et al. 2010). This finding is consistent with that of Marchant and Bossert described above because vSub is a primary output region of VH (Groenewegen et al. 1987; Naber and Witter 1998). Muscimol+baclofen inactivation of vSub decreases context-induced relapse to heroin seeking after extinction and context-induced relapse to alcohol seeking after punishment (Bossert and Stern 2014; Marchant et al. 2016). Additionally, as mentioned above, inhibition of the vSub projection to NAc shell by either pharmacological disconnection or by chemogenetic-mediated projection-specific inhibition decreases context-induced relapse to heroin seeking after extinction and context-induced relapse to alcohol seeking after punishment (Bossert et al. 2016; Marchant et al. 2016).

### Basolateral and central amygdala

In a recent study, within the same experiments and using identical training parameters in context A, we directly tested the effect of muscimol+baclofen inactivation of basolateral and central amygdala (BLA and CeA) on context-induced relapse after either extinction or punishment in context B (Pelloux et al. 2018b). We found that either BLA or CeA inactivation decreases context-induced relapse of cocaine seeking in the classical extinction ABA renewal model. However, in rats that received punishment of cocaine self-administration in context B, BLA inactivation increases context-induced relapse in context A. We found no effect of CeA inactivation on context-induced relapse after punishment. Our manipulations in context B, however, demonstrated significantly contrasting effects between rats trained for extinction versus punishment. We found that either BLA or CeA inactivation provoked relapse in context B after punishment, but not after extinction. The results of this study demonstrate dissociable roles of the two amygdala subregions in context-induced relapse after extinction versus punishment. The BLA results of our study for context-induced relapse to drug seeking after extinction are consistent with results from previous studies (Chaudhri et al. 2013; Fuchs et al. 2005; Marinelli et al. 2010; Stringfield et al. 2016). Together, these results demonstrate that the amygdala’s role in context-induced relapse critically depends on the method used to achieve abstinence.

## Conclusions and clinical implications

We reviewed the results from recent studies on context-induced relapse to alcohol and cocaine seeking after punishment-imposed abstinence and compared the results to those of previously published studies on context-induced relapse to drug seeking after extinction-imposed abstinence. As discussed above and outlined in Table 1 (Fos studies), and Table 2 and Fig. 1 (effect of site-specific neuropharmacological manipulations), there are both similarities and differences in the brain areas activated during the relapse tests after extinction versus punishment, and in the effect of different neuropharmacological manipulations on relapse in the two variations of the ABA renewal model. The LH, NAc core and shell, vSub, and the glutamatergic projection from vSub to NAc shell appear to be critical for context-induced relapse across drug classes, independent of the method used to achieve abstinence in context B. In contrast, the CeA plays a selective role in context-induced relapse to cocaine seeking after extinction but not punishment. Perhaps more significantly, the BLA appears to exert opposing control over cocaine seeking for context-induced relapse after extinction versus after punishment. A question for future research is whether the opposing roles of BLA generalize to other addictive drugs. Previous studies have shown notable differences in brain areas and projections that control context-induced relapse to heroin, cocaine, and alcohol seeking after extinction, as well as the seeking of these drugs in other relapse models (Badiani et al. 2011; Bossert et al. 2013). Below we speculate about some implications to human drug relapse of the rodent studies using the extinction- and punishment-based ABA renewal models.

We and others have previously proposed that the negative results from human “cue exposure” studies may be explained by the very reliable effect of exposure to the drug-associated context on drug seeking (reinstatement), even though the discrete cues are previously extinguished in a different context (Conklin 2006; Crombag et al. 2008). The general finding from these studies is that most human drug users relapse to drug use when they return to their home environment after successful extinction of the physiological and psychological responses to drug-associated discrete cues in the clinic (Carter and Tiffany 1999; Conklin and Tiffany 2002). In a similar manner, the results from our recent studies on context-induced relapse after punishment-imposed abstinence in a non-drug context are likely relevant to the high relapse rates in the home environment after periods of incarceration (Binswanger et al. 2013; Chandler et al. 2009; Dolan et al. 2005) or inpatient treatment (Hunt et al. 1971; Sinha 2011) where continued drug use typically results in adverse consequences like loss of privileges or verbal reprimand.

Finally, the finding that the role of the amygdala in context-induced relapse depends on the method used to achieve abstinence has implications to both animal models of relapse and human relapse. Regarding animal models, this finding highlights the importance of studying relapse under abstinence conditions that more closely mimic the human condition (Marchant et al. 2013b; Venniro et al. 2016). Regarding clinical implications, many human imaging studies reported that exposure to drug-associated cues activates the amygdala (Grant et al. 1996; Jasinska et al. 2014). The results of our recent study (Pelloux et al. 2018a) imply that the role of human amygdala in drug craving and relapse will be significantly influenced by external environmental conditions, as well as the internal motivational factors that lead to abstinence in individual drug addicts. It would be of interest to determine whether variability in the motivation for abstinence in the clinical population can reliably predict any variability observed in either the neuronal responsiveness to drug-associated cues or the propensity for relapse.

## References

[CR1] Badiani A, Belin D, Epstein D, Calu D, Shaham Y (2011). Opiate versus psychostimulant addiction: the differences do matter. Nat Rev Neurosci.

[CR2] Baker A, Steinwald H, Bouton ME (1991). Contexual conditioning and reinstatement of extinguished instrumental responding. Q J Exp Psychol.

[CR3] Binswanger IA, Blatchford PJ, Mueller SR, Stern MF (2013). Mortality after prison release: opioid overdose and other causes of death, risk factors, and time trends from 1999 to 2009. Ann Intern Med.

[CR4] Bolles RC, Holtz R, Dunn T, Hill W (1980). Comparisons of stimulus learning and response learning in a punishment situation. Learn Motiv.

[CR5] Bossert JM, Stern AL (2014). Role of ventral subiculum in context-induced reinstatement of heroin seeking in rats. Addict Biol.

[CR6] Bossert JM, Liu SY, Lu L, Shaham Y (2004). A role of ventral tegmental area glutamate in contextual cue-induced relapse to heroin seeking. J Neurosci.

[CR7] Bossert JM, Gray SM, Lu L, Shaham Y (2006). Activation of group II metabotropic glutamate receptors in the nucleus accumbens shell attenuates context-induced relapse to heroin seeking. Neuropsychopharmacology.

[CR8] Bossert JM, Poles GC, Wihbey KA, Koya E, Shaham Y (2007). Differential effects of blockade of dopamine D1-family receptors in nucleus accumbens core or shell on reinstatement of heroin seeking induced by contextual and discrete cues. J Neurosci.

[CR9] Bossert JM, Stern AL, Theberge FR, Cifani C, Koya E, Hope BT, Shaham Y (2011). Ventral medial prefrontal cortex neuronal ensembles mediate context-induced relapse to heroin. Nat Neurosci.

[CR10] Bossert JM, Marchant NJ, Calu DJ, Shaham Y (2013). The reinstatement model of drug relapse: recent neurobiological findings, emerging research topics, and translational research. Psychopharmacology.

[CR11] Bossert JM, Adhikary S, St Laurent R, Marchant NJ, Wang HL, Morales M, Shaham Y (2016). Role of projections from ventral subiculum to nucleus accumbens shell in context-induced reinstatement of heroin seeking in rats. Psychopharmacology.

[CR12] Bouton ME (1993). Context, time, and memory retrieval in the interference paradigms of Pavlovian learning. Psychol Bull.

[CR13] Bouton ME (2000). A learning theory perspective on lapse, relapse, and the maintenance of behavior change. Health Psychol.

[CR14] Bouton ME (2002). Context, ambiguity, and unlearning: sources of relapse after behavioral extinction. Biol Psychiatry.

[CR15] Bouton ME, Bolles RC (1979). Role of conditioned contextual stimuli in reinstatement of extinguished fear. J Exp Psychol Anim Behav Process.

[CR16] Bouton ME, Schepers ST (2015). Renewal after the punishment of free operant behavior. J Exp Psychol Anim Learn Cogn.

[CR17] Bouton ME, Todd TP (2014). A fundamental role for context in instrumental learning and extinction. Behav Process.

[CR18] Carter BL, Tiffany ST (1999). Meta-analysis of cue-reactivity in addiction research. Addiction.

[CR19] Chandler RK, Fletcher BW, Volkow ND (2009). Treating drug abuse and addiction in the criminal justice system: improving public health and safety. JAMA.

[CR20] Chaudhri N, Sahuque LL, Cone JJ, Janak PH (2008). Reinstated ethanol-seeking in rats is modulated by environmental context and requires the nucleus accumbens core. Eur J Neurosci.

[CR21] Chaudhri N, Sahuque LL, Janak PH (2009). Ethanol seeking triggered by environmental context is attenuated by blocking dopamine D1 receptors in the nucleus accumbens core and shell in rats. Psychopharmacology.

[CR22] Chaudhri N, Sahuque LL, Schairer WW, Janak PH (2010). Separable roles of the nucleus accumbens core and shell in context- and cue-induced alcohol-seeking. Neuropsychopharmacology.

[CR23] Chaudhri N, Woods CA, Sahuque LL, Gill TM, Janak PH (2013). Unilateral inactivation of the basolateral amygdala attenuates context-induced renewal of Pavlovian-conditioned alcohol-seeking. Eur J Neurosci.

[CR24] Conklin CA (2006). Environments as cues to smoke: implications for human extinction-based research and treatment. Exp Clin Psychopharmacol.

[CR25] Conklin CA, Tiffany ST (2002). Applying extinction research and theory to cue-exposure addiction treatments. Addiction.

[CR26] Crombag HS, Shaham Y (2002). Renewal of drug seeking by contextual cues after prolonged extinction in rats. Behav Neurosci.

[CR27] Crombag HS, Grimm JW, Shaham Y (2002). Effect of dopamine receptor antagonists on renewal of cocaine seeking by reexposure to drug-associated contextual cues. Neuropsychopharmacology.

[CR28] Crombag HS, Bossert JM, Koya E, Shaham Y (2008). Review. Context-induced relapse to drug seeking: a review. Philos Trans R Soc Lond Ser B Biol Sci.

[CR29] Cruz FC, Koya E, Guez-Barber DH, Bossert JM, Lupica CR, Shaham Y, Hope BT (2013). New technologies for examining the role of neuronal ensembles in drug addiction and fear. Nat Rev Neurosci.

[CR30] Cruz FC, Babin KR, Leao RM, Goldart EM, Bossert JM, Shaham Y, Hope BT (2014). Role of nucleus accumbens shell neuronal ensembles in context-induced reinstatement of cocaine-seeking. J Neurosci.

[CR31] de Wit H, Stewart J (1981). Reinstatement of cocaine-reinforced responding in the rat. Psychopharmacology.

[CR32] Deroche-Gamonet V, Belin D, Piazza PV (2004). Evidence for addiction-like behavior in the rat. Science.

[CR33] Diergaarde L, de Vries W, Raaso H, Schoffelmeer AN, De Vries TJ (2008). Contextual renewal of nicotine seeking in rats and its suppression by the cannabinoid-1 receptor antagonist rimonabant (SR141716A). Neuropharmacology.

[CR34] Dolan KA, Shearer J, White B, Zhou J, Kaldor J, Wodak AD (2005). Four-year follow-up of imprisoned male heroin users and methadone treatment: mortality, re-incarceration and hepatitis C infection. Addiction.

[CR35] Epstein DH, Preston KL (2003). The reinstatement model and relapse prevention: a clinical perspective. Psychopharmacology.

[CR36] Epstein DH, Preston KL, Stewart J, Shaham Y (2006). Toward a model of drug relapse: an assessment of the validity of the reinstatement procedure. Psychopharmacology.

[CR37] Estes WK, Skinner BF (1941). Some quantitative properties of anxiety. J Exp Psychol.

[CR38] Fuchs RA, Evans KA, Ledford CC, Parker MP, Case JM, Mehta RH, See RE (2005). The role of the dorsomedial prefrontal cortex, basolateral amygdala, and dorsal hippocampus in contextual reinstatement of cocaine seeking in rats. Neuropsychopharmacology.

[CR39] Fuchs RA, Eaddy JL, Su ZI, Bell GH (2007). Interactions of the basolateral amygdala with the dorsal hippocampus and dorsomedial prefrontal cortex regulate drug context-induced reinstatement of cocaine-seeking in rats. Eur J Neurosci.

[CR40] Fuchs R, Ramirez D, Bell G (2008). Nucleus accumbens shell and core involvement in drug context-induced reinstatement of cocaine seeking in rats. Psychopharmacology.

[CR41] Gold RM (1966). Aphagia and adipsia produced by unilateral hypothalamic lesions in rats. Am J Phys.

[CR42] Grant S, London ED, Newlin DB, Villemagne VL, Liu X, Contoreggi C, Phillips RL, Kimes AS, Margolin A (1996). Activation of memory circuits during cue-elicited cocaine craving. Proc Natl Acad Sci U S A.

[CR43] Groenewegen HJ, Vermeulen-Van der Zee E, te Kortschot A, Witter MP (1987). Organization of the projections from the subiculum to the ventral striatum in the rat. A study using anterograde transport of *Phaseolus vulgaris* leucoagglutinin. Neuroscience.

[CR44] Hamlin AS, Newby J, McNally GP (2007). The neural correlates and role of D1 dopamine receptors in renewal of extinguished alcohol-seeking. Neuroscience.

[CR45] Hamlin AS, Clemens KJ, McNally GP (2008). Renewal of extinguished cocaine-seeking. Neuroscience.

[CR46] Hamlin AS, Clemens KJ, Choi EA, McNally GP (2009). Paraventricular thalamus mediates context-induced reinstatement (renewal) of extinguished reward seeking. Eur J Neurosci.

[CR47] Holland PC, Medlin DL (1992). Occasion setting in Pavlovian conditioning. Psychology of learning and motivation.

[CR48] Hunt WA, Barnett LW, Branch LG (1971). Relapse rates in addiction programs. J Clin Psychol.

[CR49] Jasinska AJ, Stein EA, Kaiser J, Naumer MJ, Yalachkov Y (2014). Factors modulating neural reactivity to drug cues in addiction: a survey of human neuroimaging studies. Neurosci Biobehav Rev.

[CR50] Jean-Richard-Dit-Bressel P, McNally GP (2015). The role of the basolateral amygdala in punishment. Learn Mem.

[CR51] Jean-Richard-Dit-Bressel P, Killcross S, McNally GP (2018) Behavioural and neurobiological mechanisms of punishment: implications for psychiatric disorders. Neuropsychopharmacology in press 10.1038/s41386-018-0047-3 PMC6006171 29703994

[CR52] Katz JL, Higgins ST (2003). The validity of the reinstatement model of craving and relapse to drug use. Psychopharmacology.

[CR53] Koya E, Golden SA, Harvey BK, Guez-Barber DH, Berkow A, Simmons DE, Bossert JM, Nair SG, Uejima JL, Marin MT, Mitchell TB, Farquhar D, Ghosh SC, Mattson BJ, Hope BT (2009). Targeted disruption of cocaine-activated nucleus accumbens neurons prevents context-specific sensitization. Nat Neurosci.

[CR54] Lasseter HC, Xie X, Ramirez DR, Fuchs RA (2010). Sub-region specific contribution of the ventral hippocampus to drug context-induced reinstatement of cocaine-seeking behavior in rats. Neuroscience.

[CR55] Marchant NJ, Kaganovsky K (2015). A critical role of nucleus accumbens dopamine D1-family receptors in renewal of alcohol seeking after punishment-imposed abstinence. Behav Neurosci.

[CR56] Marchant NJ, Hamlin AS, McNally GP (2009). Lateral hypothalamus is required for context-induced reinstatement of extinguished reward seeking. J Neurosci.

[CR57] Marchant NJ, Furlong TM, McNally GP (2010). Medial dorsal hypothalamus mediates the inhibition of reward seeking after extinction. J Neurosci.

[CR58] Marchant NJ, Khuc TN, Pickens CL, Bonci A, Shaham Y (2013). Context-induced relapse to alcohol seeking after punishment in a rat model. Biol Psychiatry.

[CR59] Marchant NJ, Li X, Shaham Y (2013). Recent developments in animal models of drug relapse. Curr Opin Neurobiol.

[CR60] Marchant NJ, Rabei R, Kaganovsky K, Caprioli D, Bossert JM, Bonci A, Shaham Y (2014). A critical role of lateral hypothalamus in context-induced relapse to alcohol seeking after punishment-imposed abstinence. J Neurosci.

[CR61] Marchant NJ, Kaganovsky K, Shaham Y, Bossert JM (2015). Role of corticostriatal circuits in context-induced reinstatement of drug seeking. Brain Res.

[CR62] Marchant NJ, Campbell EJ, Whitaker LR, Harvey BK, Kaganovsky K, Adhikary S, Hope BT, Heins RC, Prisinzano TE, Vardy E, Bonci A, Bossert JM, Shaham Y (2016). Role of ventral subiculum in context-induced relapse to alcohol seeking after punishment-imposed abstinence. J Neurosci.

[CR63] Marchant NJ, Campbell EJ, Kaganovsky K (2017) Punishment of alcohol-reinforced responding in alcohol preferring P rats reveals a bimodal population: implications for models of compulsive drug seeking. Prog Neuro-Psychopharmacol Biol Psychiatry. 10.1016/j.pnpbp.2017.07.020 10.1016/j.pnpbp.2017.07.020 PMC5785579 28754407

[CR64] Marinelli PW, Funk D, Juzytsch W, Le AD (2010). Opioid receptors in the basolateral amygdala but not dorsal hippocampus mediate context-induced alcohol seeking. Behav Brain Res.

[CR65] Marlatt GA (2002). Do animal models provide a valid analogue for human drug lapse and relapse? Comment on Leri and Stewart (2002). Exp Clin Psychopharmacol.

[CR66] McGlinchey EM, Aston-Jones G (2018). Dorsal hippocampus drives context-induced cocaine seeking via inputs to lateral septum. Neuropsychopharmacology.

[CR67] Millan EZ, McNally GP (2011). Accumbens shell AMPA receptors mediate expression of extinguished reward seeking through interactions with basolateral amygdala. Learn Mem.

[CR68] Millan EZ, McNally GP (2012). Cocaine- and amphetamine-regulated transcript in the nucleus accumbens shell attenuates context-induced reinstatement of alcohol seeking. Behav Neurosci.

[CR69] Millan EZ, Furlong TM, McNally GP (2010). Accumbens shell-hypothalamus interactions mediate extinction of alcohol seeking. J Neurosci.

[CR70] Millan EZ, Marchant NJ, McNally GP (2011). Extinction of drug seeking. Behav Brain Res.

[CR71] Morgan JI, Curran T (1991). Stimulus-transcription coupling in the nervous system: involvement of the inducible proto-oncogenes fos and jun. Annu Rev Neurosci.

[CR72] Naber PA, Witter MP (1998). Subicular efferents are organized mostly as parallel projections: a double-labeling, retrograde-tracing study in the rat. J Comp Neurol.

[CR73] Nakajima S, Tanaka S, Urushihara K, Imada H (2000). Renewal of extinguished lever-press responses upon return to the training context. Learn Motiv.

[CR74] O'Brien CP, Childress AR, McLellan AT, Ehrman R (1992). Classical conditioning in drug-dependent humans. Ann N Y Acad Sci.

[CR75] Palombo P, Leao RM, Bianchi PC, de Oliveira PEC, Planeta CDS, Cruz FC (2017). Inactivation of the prelimbic cortex impairs the context-induced reinstatement of ethanol seeking. Front Pharmacol.

[CR76] Panlilio L, Thorndike E, Schindler C (2003). Reinstatement of punishment-suppressed opioid self-administration in rats: an alternative model of relapse to drug abuse. Psychopharmacology.

[CR77] Pelloux Y, Everitt B, Dickinson A (2007). Compulsive drug seeking by rats under punishment: effects of drug taking history. Psychopharmacology.

[CR78] Pelloux Y, Hoots JK, Cifani C, Adhikary S, Martin J, Minier-Toribio A, Bossert JM, Shaham Y (2018). Context-induced relapse to cocaine seeking after punishment-imposed abstinence is associated with activation of cortical and subcortical brain regions. Addict Biol.

[CR79] Pelloux Y, Minier-Toribio A, Hoots JK, Bossert JM, Shaham Y (2018). Opposite effects of basolateral amygdala inactivation on context-induced relapse to cocaine seeking after extinction versus punishment. J Neurosci.

[CR80] Perry CJ, McNally GP (2013). mu-Opioid receptors in the nucleus accumbens shell mediate context-induced reinstatement (renewal) but not primed reinstatement of extinguished alcohol seeking. Behav Neurosci.

[CR81] Peters J, Vallone J, Laurendi K, Kalivas PW (2008). Opposing roles for the ventral prefrontal cortex and the basolateral amygdala on the spontaneous recovery of cocaine-seeking in rats. Psychopharmacology.

[CR82] Piantadosi PT, Yeates DCM, Wilkins M, Floresco SB (2017). Contributions of basolateral amygdala and nucleus accumbens subregions to mediating motivational conflict during punished reward-seeking. Neurobiol Learn Mem.

[CR83] Pickens CL, Golden SA, Adams-Deutsch T, Nair SG, Shaham Y (2009). Long-lasting incubation of conditioned fear in rats. Biol Psychiatry.

[CR84] Prasad AA, McNally GP (2016). Ventral pallidum output pathways in context-induced reinstatement of alcohol seeking. J Neurosci.

[CR85] Rubio FJ, Liu QR, Li X, Cruz FC, Leao RM, Warren BL, Kambhampati S, Babin KR, McPherson KB, Cimbro R, Bossert JM, Shaham Y, Hope BT (2015). Context-induced reinstatement of methamphetamine seeking is associated with unique molecular alterations in Fos-expressing dorsolateral striatum neurons. J Neurosci.

[CR86] Shaham Y, Shalev U, Lu L, de Wit H, Stewart J (2003). The reinstatement model of drug relapse: history, methodology and major findings. Psychopharmacology.

[CR87] Sinha R (2011). New findings on biological factors predicting addiction relapse vulnerability. Curr Psychiatry Rep.

[CR88] Stringfield SJ, Higginbotham JA, Fuchs RA (2016). Requisite role of basolateral amygdala glucocorticoid receptor stimulation in drug context-induced cocaine-seeking behavior. Int J Neuropsychopharmacol.

[CR89] Todd TP, Vurbic D, Bouton ME (2014). Behavioral and neurobiological mechanisms of extinction in Pavlovian and instrumental learning. Neurobiol Learn Mem.

[CR90] Vanderschuren LJMJ, Everitt BJ (2004). Drug seeking becomes compulsive after prolonged cocaine self-administration. Science.

[CR91] Vanderschuren LJM, Minnaard AM, Smeets JAS, Lesscher HMB (2017). Punishment models of addictive behavior. Curr Opin Behav Sci.

[CR92] Venniro M, Caprioli D, Shaham Y (2016). Animal models of drug relapse and craving: from drug priming-induced reinstatement to incubation of craving after voluntary abstinence. Prog Brain Res.

[CR93] Widholm JJ, Gass JT, Cleva RM, Olive MF (2011) The mGluR5 positive allosteric modulator CDPPB does not alter extinction or contextual reinstatement of methamphetamine-seeking behavior in rats. J Addict Res Ther. 10.4172/2155-6105.S1-004 10.4172/2155-6105.S1-004 PMC3305267 22428090

[CR94] Wikler A (1973). Dynamics of drug dependence. Implications of a conditioning theory for research and treatment. Arch Gen Psychiatry.

[CR95] Wolffgramm J, Heyne A (1995). From controlled drug intake to loss of control: the irreversible development of drug addiction in the rat. Behav Brain Res.

[CR96] Xie X, Lasseter HC, Ramirez DR, Ponds KL, Wells AM, Fuchs RA (2012). Subregion-specific role of glutamate receptors in the nucleus accumbens on drug context-induced reinstatement of cocaine-seeking behavior in rats. Addict Biol.

